# Implementation and sustainment of a statewide telemedicine diabetic retinopathy screening network for federally designated safety-net clinics

**DOI:** 10.1371/journal.pone.0241767

**Published:** 2020-11-04

**Authors:** Ana Bastos de Carvalho, S. Lee Ware, Feitong Lei, Heather M. Bush, Robert Sprang, Eric B. Higgins

**Affiliations:** 1 Department of Ophthalmology and Visual Sciences, University of Kentucky College of Medicine, Lexington, Kentucky, United States of America; 2 Department of Biostatistics, University of Kentucky College of Public Health, Lexington, Kentucky, United States of America; 3 Kentucky Telecare, University of Kentucky College of Medicine, Lexington, Kentucky, United States of America; Drexel University School of Public Health, UNITED STATES

## Abstract

**Context:**

Diabetic retinopathy (DR) is the leading cause of incident blindness among working-age adults in the United States. Federally designated safety-net clinics (FDSC) often serve as point-of-contact for patients least likely to receive recommended DR screenings, creating opportunity for targeted interventions to increase screening access and compliance.

**Study design and methods:**

With such a goal, we implemented and assessed the longitudinal performance of an FDSC-based telemedicine DR screening (TDRS) network of 22 clinical sites providing nonmydriatic fundus photography with remote interpretation and reporting. Retrospective analysis of patient encounters between February 2014 and January 2019 was performed to assess rates of pathology and referral. A generalized estimating equation logistic regression model was used for subset analysis from audits of pre- and post-implementation screening rates. Finally, patient surveys were conducted and assessed as a measure of intervention acceptability.

**Results:**

Of the 13,923 individual telescreening encounters (4327 female, 4220 male, and 5376 unspecified; mean [SD] age, 54.9 [12.5] years) studied, 10,540 were of adequate quality to identify 3532 (33.5%) patients with ocular pathology: 2319 (22.0%) patients had some level of DR with 1604 (15.2%) requiring specialist referral, and 808 (7.7%) patients required referral for other ocular pathologies. The mean screening rate for audited clinics in the year prior to program implementation was 29.9% (641/2147), which increased to 47.7% (1012/2124) in the program’s first year, doubling patients’ odds of being screened (OR 2.2; 95% CI: 1.3–3.7; *P* = .003). These gains were sustained over four years following implementation (OR 1.9; 95% CI: 1.1–3.1; *P* = .018) despite varied clinic screening performance (4-year averaged range, 22.9–55.1%). Odds of early detection likewise doubled for patients with consecutive screenings (OR 2.2, 95% CI: 2.0–2.4; *P* < .001). Finally, surveyed patients preferred TDRS to specialist exams (82.5%; 776/941) and would recommend the service to friends (92.7%; 868/936).

**Conclusion and relevance:**

A statewide, FDSC-centered TDRS network was successfully established and sustained in a medically underserved region of the United States. Our results suggest that large TDRS networks in FDSCs can increase screening access and compliance for otherwise unscreened populations, but outcomes can vary greatly among clinics. Further work to optimize program implementation is needed to maximize this model’s impact.

## Introduction

Diabetic retinopathy (DR), a common microvascular complication of diabetes mellitus, is the leading cause of incident blindness among working-age adults [1]. Annual DR screening with early intervention can reduce the risk of severe DR-related vision loss by 90% [2–7] and is recommended by the American Academy of Ophthalmology for all diabetic patients [8]. Yet DR screening rates in the US remain low [9,10], especially among vulnerable populations [11–14]. These missed opportunities for secondary prevention decrease productivity [15], increase morbidity [16,17], and increase healthcare expenditures [18,19]. With effective treatments for DR available, the more intractable challenge has been consistent screening for early identification of those requiring closer monitoring or therapeutic intervention.

An estimated 88% of diabetic patients in the US visit their primary care provider (PCP) at least once per year [20], creating a consistent point of contact for leveraging nonmydriatic fundus photography (nFP) and cloud-based telemedicine to provide DR screenings embedded in the primary care setting [21]. Telemedicine DR screening (TDRS) may mitigate several of the known barriers to conventional DR exams, including socioeconomic factors [22–24], geographic and logistic obstacles [22,25], lack of motivation [22,25], absence of PCP referral [22,26], inconveniences associated with pupil dilation [27], and specialist availability [24,25]. Images taken in the primary care setting during provision of routine care can be uploaded to remote specialists for interpretation and as-needed referral. This evidence-based intervention (EBI) [28] has high sensitivity and specificity,[29,30] making it clinically comparable to the criterion standard for DR screening (in-person specialist-performed dilated fundus exam) [21,22], and superior to direct ophthalmoscopy performed by PCPs [28,31,32].

Federally designated safety-net clinics (FDSC), including Federally Qualified Health Centers (FQHC) and rural health clinics (RHC), are cornerstones of the primary care safety-net in the United States (US), providing vulnerable populations with primary health services and care coordination. FDSCs are often the only accessible point of care for underserved diabetic patients [33,34]. To our knowledge, the effectiveness of the TDRS model to increase DR screening rates in a statewide network of FDSCs exposed to the diverse patient and payer dynamics of the US health system has not been explored.

With these facts in mind, we began building a statewide TDRS network in 2013. The partnership connected FDSCs across Kentucky to an academic medical center providing low-cost telemedicine services (image interpretation, reporting, and referral). We herein present and analyze the network’s implementation process and results, including surveillance data on rates of DR and non-DR pathologies and effects of continuity of care, from the first five years of the program. Further, we assess pre- and post-implementation screening rates from a subset of clinic sites and discuss results of patient surveys intended to assess intervention acceptability.

## Methods

The Appalachia Diabetic Eye Network (ADEN) was established as a blindness prevention initiative to extend DR screening access for underserved populations in Kentucky via primary care-embedded telemedicine. A collaboration between the Department of Ophthalmology and Visual Sciences at the University of Kentucky (UKY) and multiple FDSCs across the state, the ADEN reaches both rural and urban populations.

Both national and state-targeted needs assessments were conducted to establish the justification and intervention strategy for the program, and findings were crosswalked to objectives defined by the World Health Organization’s Healthy People 2020 initiative.

This study conformed to the tenets of the Declaration of Helsinki, was approved by the University of Kentucky’s institutional review board, and was conducted in compliance with the Health Insurance Portability and Accountability Act.

### TDRS program implementation

#### Clinic recruitment and program integration

During the Pilot phase, FDSC administrators were contacted by a program director (RS) and invited to participate in the program. Clinics were offered nFP cameras, interpretation and reporting services, staff training, and ongoing technical support free of charge for three years (through grant funding), after which a nominal fee was charged per screening for interpretation.

An unfunded Expansion phase was opened to meet demand. Clinics were required to independently finance the purchase of cameras and were charged the interpretation fee beginning with their first screening.

The same model nFP camera (Centervue DRS^®^ desktop nonmydriatic autofocus camera; Centervue S.p.A, Padova, Italy) was used by all participating clinics of both phases.

As network study sites were recruited, retrospective baseline data was collected from each regarding their diabetic patient population. Patients were surveyed to assess pre-implementation patient-perspective attitudes regarding the proposed intervention.

**Fig 1:**
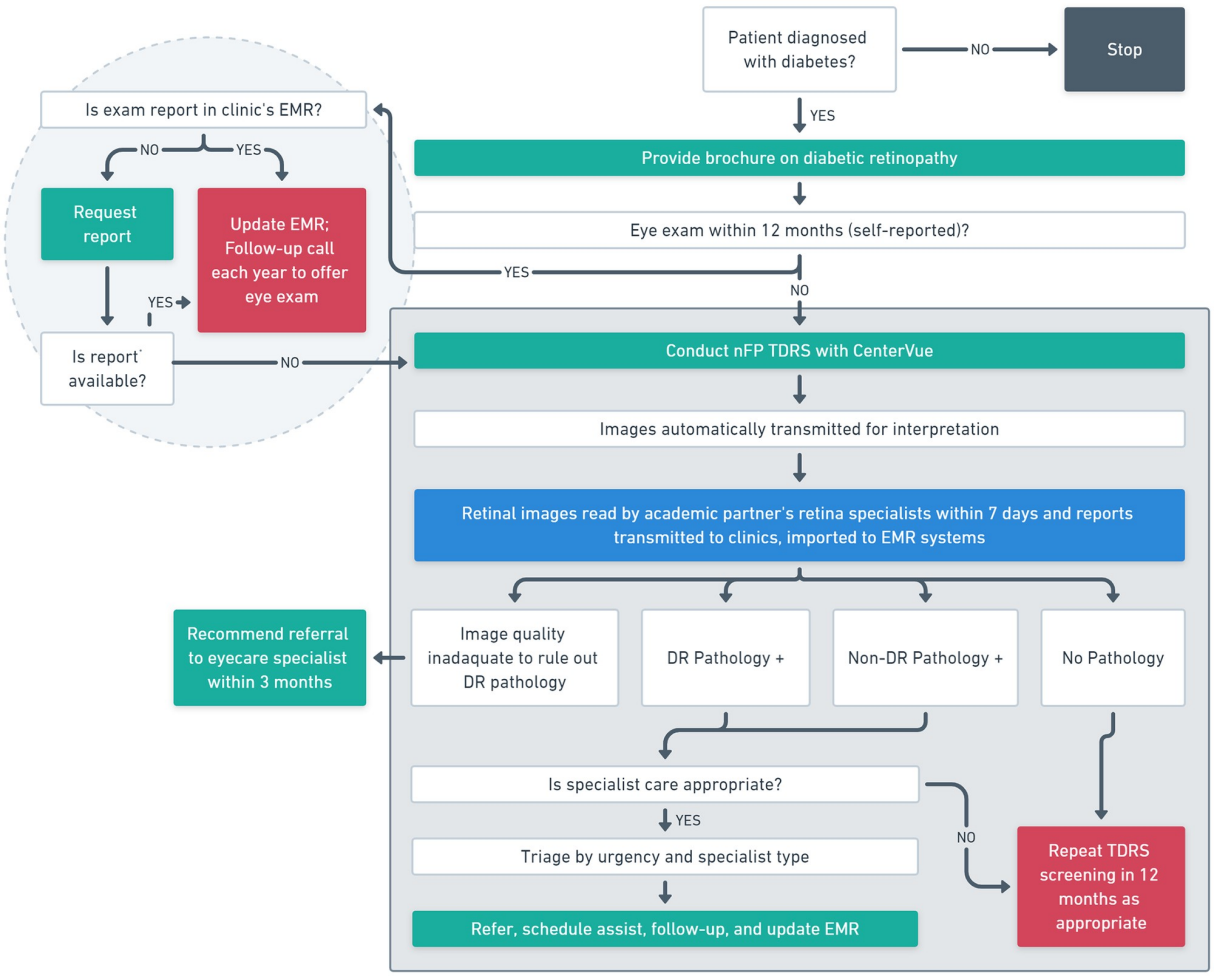
Appalachia Diabetic Retinopathy Network TDRS Clinical Workflow Model. The clinical workflow distributed to ADEN sites begins with the identification of diabetic patients, and walks through the key questions (white), actions (green), and end-points (red) to be completed for each encounter. Abbreviations: TDRS, Telemedicine diabetic retinopathy screening; EMR, Electronic medical record; nFP, Nonmydriatic fundus photography; DR, Diabetic retinopathy.

Participating FDSCs were independent or belonged to multi-clinic systems. In some cases, all clinics in an FDSC system participated in the program, but often not. Such decisions were left to FDSC leadership and based on the resource constraints, priorities, patient needs, and provider buy-in idiosyncratic to each site. A model workflow (Fig 1) was developed by program directors and distributed to each administration as part of the implementation package of resources.

To accommodate the heterogeneous administrative and workflow characteristics of network sites, TDRS integration was intentionally made flexible, allowing each site latitude in adapting the protocols and processes that would govern day-to-day operation of the TDRS service.

During the study period, all FDSCs provided TDRS to patients as a free service (i.e., not billed to insurance and triggered no out-of-pocket cost) since cost of care is among the most significant patient-perceived barriers to TDRS utilization [22].

#### Patient recruitment

We performed a retrospective electronic chart review of all patients who participated in the ADEN program between February 01, 2014 and January 31, 2019. According to the screening protocol, all nonpregnant adults with types 1 or 2 diabetes and without a complete eye exam or DR screening documented during the prior 12 months should be offered TDRS in clinic during regularly scheduled appointments (Fig 1).

### Imaging

Pre-existing clinic staff were trained to use the screening protocol (Fig 1). nFP training was completed in single sessions simultaneous with installation of the camera systems on site by company (Centervue) representatives and trainers.

Staff performed image captures using a 45° field of view centered between the optic disc and fovea (Central Nasal) to include the optic disc, macula, and vascular arcades. One image per eye was uploaded to a secure cloud storage server. The interpretations were performed by the partner institution (UKY) utilizing REDCap software (Research Electronic Data Capture; Vanderbilt University, Nashville, Tennessee, USA).

### Image interpretation

Images were reviewed and interpreted by UKY retina specialists without access to clinics’ electronic medical record (EMR) systems. Specialists noted whether each image was gradable or ungradable, the absence or presence and severity of DR, the absence or presence of diabetic macular edema, as well as findings concerning for nondiabetic pathologies considered by the grading ophthalmologist to be life- or sight-threatening. DR severity was graded according to the International Clinical Disease Severity Scales for DR and DME (Table 1) [35]. When evidence of DR was observed in both eyes, the patient’s overall severity was defined by the more severe grade. If at least one image showed referable pathology, a referral for that purpose was made. An image was considered ungradable if both 1) no signs of pathology were discernible in viewable areas, and 2) subtle pathology could not be ruled out for at least 60% of the macula. The program met the American Telemedicine Association’s criteria for Validation Category 3 [36].

**Table 1:** International Clinical Diabetic Retinopathy and Diabetic Macular Edema Disease Severity Scales.

Classification	Observable Findings
No apparent retinopathy	No abnormalities
Mild nonproliferative diabetic retinopathy	Microaneurysms only
Moderate nonproliferative diabetic retinopathy	More than just microaneurysms but less than severe nonproliferative diabetic retinopathy
Severe nonproliferative diabetic retinopathy	Any of the following: more than 20 intraretinal hemorrhages in each of 4 quadrants; definite venous beading in 2+ quadrants; Prominent intraretinal microvascular abnormalities in 1+ quadrant *and no* signs of proliferative retinopathy
Proliferative diabetic retinopathy	One or more of the following: neovascularization, vitreous/preretinal hemorrhage
No apparent macular edema	No apparent retinal thickening or hard exudates in posterior pole
Diabetic macular edema is present	Some apparent retinal thickening or hard exudates in posterior pole

### Reading center reporting and recommendations

Reports with follow-up and referral recommendations were sent to point-of-care clinics. Referrals were based on the presence and level of DR, the presence of non-DR findings (or both, when appropriate), and if one or both of the images were ungradable.

Patients without pathology or with mild nonproliferative diabetic retinopathy (NPDR) were recommended for repeat exam in 12 months. Patients with moderate NPDR were referred to an eye care provider to be seen within 3 months, while those with severe NPDR, proliferative DR, or diabetic macular edema (DME) were referred to a retina specialist to be seen within 1 month. Urgency of referral for other pathologies was determined case-by-case. For patients with ungradable or missing images, a referral for dilated ocular examination by an eye care specialist within three months was recommended.

### Screening rates

Pre- and post-implementation screening rates were provided by a subgroup of six Pilot phase clinics. Screening rates per year were calculated using clinic electronic medical record (EMR) queries as: number of diabetic patients with documented DR screening within 12 months / total number of diabetic patients with at least one clinical encounter during the same period.

### Patient survey

Patient acceptability of TDRS was assessed using a questionnaire offered to all screened patients at Pilot phase clinics between March 2014 and March 2017, along with a waiver of informed consent. Patient participation was voluntary. The instrument was developed by program directors (RS and EBH) and reviewed by program staff for clarity. The survey instrument was optionally presented in paper and web-based modes depending on clinic and patient preference(s), captured no identifying information, and was submitted anonymously. Electronic versions were uploaded directly and stored using REDCap software on UKY secure servers; paper versions were shredded after being transcoded to the research database.

### Analysis

Descriptive analyses were conducted for frequencies and percentages of pathologies. Because pathology could not be ruled out for ungradable images, the screening positive rate was calculated using the number of adequate screenings in the denominator, which may provide a more accurate estimate of disease burden in the screened population.

A univariate logistic regression model was used to determine the odds ratios (ORs) and 95% confidence intervals (CIs) for the association between patients’ age and adequate imaging (gradeability). A generalized estimating equation logistic regression model was used to analyze repeated screening rate measures for pre- and post-intervention periods. From patient surveys, patients’ self-reported characteristics and satisfaction responses were analyzed, with p-values calculated using Chi-square and Fisher’s exact tests, as appropriate. Missing observations were excluded on an analysis-by-analysis basis. Statistical significance was determined as p-values < .05 (two-tailed) for all tests. All analyses were performed using Statistical Analysis Software (SAS), version 9.4 (SAS Institute, Cary, NC, USA).

## Results

### Program implementation

Two FDSC systems constituting 11 clinical sites were initially recruited and signed grant contracts. All sites received the implementation package, nFP cameras, and image interpretation services as the Pilot phase cohort of clinics (Table 2). Subsequently, 11 more FDSC clinics petitioned for participation in the network. All qualified and were included in the Expansion phase (Table 2). Over the period studied, all 22 clinics remained in the network, registering a 100% clinic retention rate, and all clinics continued to offer the service free-of-charge to patients throughout and following the study period. In many clinics, costs were offset by gains in performance-based bundled-care bonuses.

**Table 2:** Timeline of TDRS clinic rollout.

Clinic Site	Study Phase	Rollout Date
Clinics 1–4	Pilot	February 2014
Clinics 5–7	Pilot	November 2014
Clinic 8	Expansion	May 2015
Clinic 9	Expansion	August 2015
Clinics 10, 11	Pilot	December 2015
Clinics 12, 13	Pilot	February 2016
Clinic 14	Expansion	February 2016
Clinic 15	Expansion	August 2016
Clinics 16, 17	Expansion	June 2017
Clinics 18–22	Expansion	December 2017

Abbreviations: TDRS, Telemedicine diabetic retinopathy screening.

This network of 22 FDSCs was comprised of 6 FQHC and 1 RHC multi-site systems, with an average of 3 participating clinics each, and 1 single-site FQHC. Clinics averaged 5.9 providers per site (range: 3–12), and all but the 5 urban sites served rural populations. All clinics had EMR systems and broadband access.

### Demographics

During the program’s first five years, a total of 13,923 TDRS exams were completed (Table 3), serving 10,056 unique patients (dataset available as a S1 Dataset). Mean patient age was 54.9 years (SD 12.5 for 13,767 records), and gender was evenly balanced (male 49.4%, female 50.6% of 8547 records).

**Table 3:** Number of TDRS encounters by year.

Year	Frequency	Proportion (%)
2014^a^	656	4.71
2015	2,061	14.80
2016	3,243	23.29
2017	3,540	25.43
2018	3,860	27.72
2019^b^	563	4.04
**Total**	**13,923**	**100**

^a^Network screening began with 4 clinics in February 2014.

^b^Cutoff for analysis was January 31st 2019.

Abbreviations: TDRS, Telemedicine diabetic retinopathy screening.

### Gradeability

Of the 13,923 exams performed, 10,540 were of sufficient quality to either rule out DR, or identify a life- or sight-threatening pathology requiring referral. We therefore report a practical gradeability (adequacy) rate of 75.7%, with annual gradable exam rates among individual clinics ranging between 65.7% and 92.7%. As expected, a significant correlation was observed between odds of gradeability and age. Probability that a screening would result in referral due to ungradable image(s) increased 5% for every 1 year increase in patient age (odds ratio: 1.1; 95% CI: 1.0–1.1).

### Pathology

Sixty-six percent (7008/10,540) of adequate exams had no discernible pathology. Diabetic retinopathy was identified in 22.0% of adequate exams (2319/10,540; 2276 with DR and 536 with DME, 499 of which overlapped) (Table 4). When defined by degree of retinopathy in the worst affected eye, 66.9% (1523/2276) of those with DR had mild NPDR; 18.1% (411/2276) had moderate NPDR; 10.5% (238/2,276) had severe NPDR; and 4.6% (104/2276) had proliferative DR. DME was present in 5.1% (536/10,540) of adequate screenings.

**Table 4:** Presence and severity of diabetic eye disease.

Disease	Total Exams (*N* = 13,923), *n* (%)	Adequate Exams (*N* = 10,540), %	DR-Positive Exams (*N* = 2319), %
No DR	8221 (59.05)	78.0	
Any DR	2276 (16.35)	21.6	98.2
Mild NPDR	1523 (10.94)	14.5	65.7
Moderate NPDR	411 (2.95)	3.9	17.7
Severe NPDR	238 (1.71)	2.3	10.3
Proliferative DR	104 (0.75)	1.0	4.5
Macular Edema	536 (3.85)	5.1	23.1

Abbreviations: DR, Diabetic retinopathy; NPDR, Nonproliferative diabetic retinopathy.

**Fig 2:**
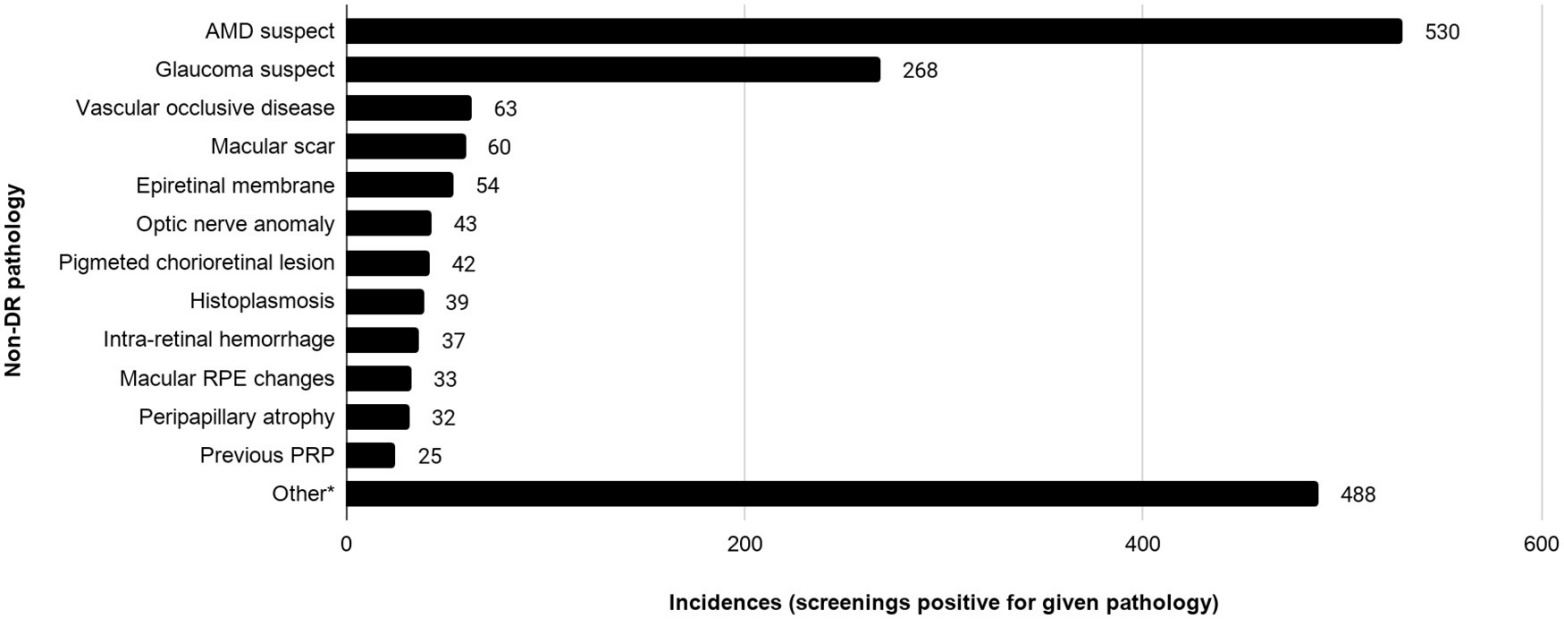
Selected incidental findings. *Other less common findings included cataract or media opacity, macular dystrophy, Hollenhorst plaque, macular hole, crystalline maculopathy, choroid scar, myelinated nerve fiber layer, asteroid hyalosis or vitreous debris, hypertensive retinopathy, myopic degeneration, optic nerve head elevation, retinal degeneration, and more. Abbreviations: AMD, Age-related macular degeneration; RPE, Retinal pigment epithelium.

Incidental findings nonspecific to diabetes were observed in 11.5% (1213/10,540) of adequate screenings. AMD suspect (5.0%; 530/10,540) and glaucomatous findings (2.5%; 268/10,540) were most commonly observed, followed by vascular occlusion (0.6%), macular scarring (0.6%), and epiretinal membrane (0.5%) (Fig 2).

### Referrals

**Fig 3:**
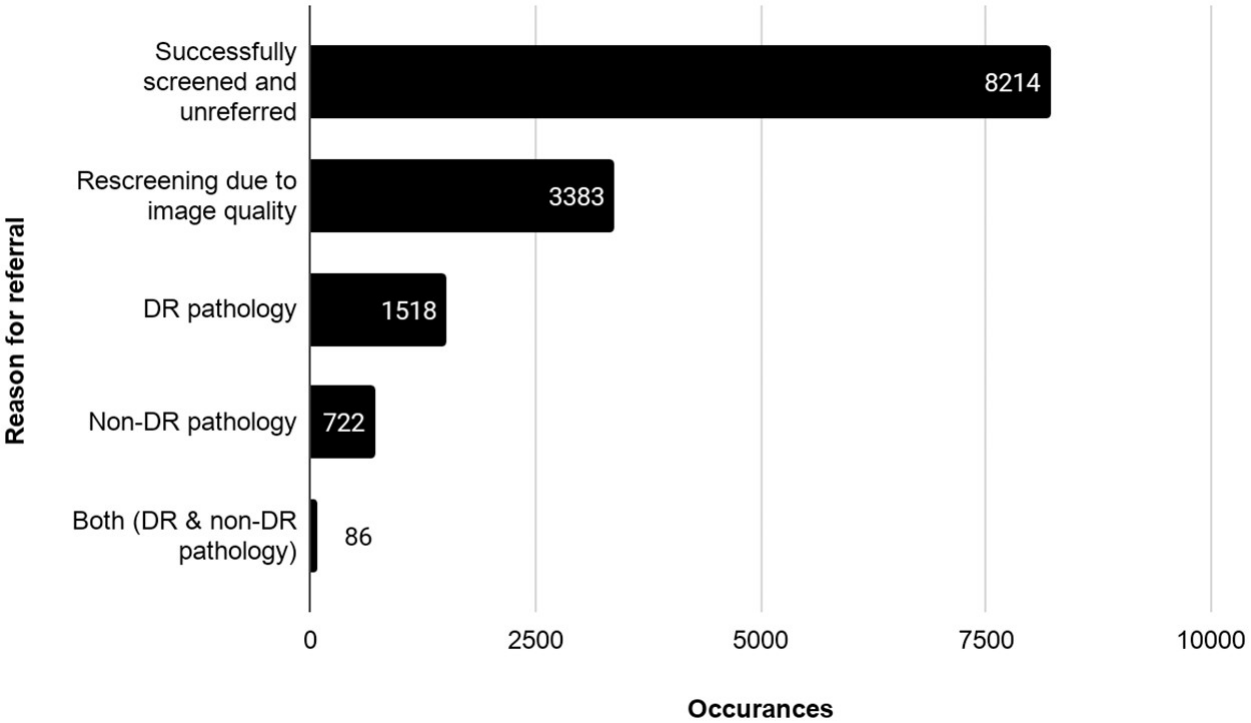
Reasons for referral to an eye care provider. Abbreviations: DR, Diabetic retinopathy.

In total, 5709 patients were recommended for follow-up or specialist referral (Fig 3). The overall rate of referral was 41.0% (5709 of 13,923 exams). The most common reason for referral was insufficient image quality (59.3%; 3383/5709), followed by diabetic pathology (28.1%; 1604/5709) and non-diabetic pathology (14.2%; 808/5709), with 86 overlapping.

**Fig 4:**
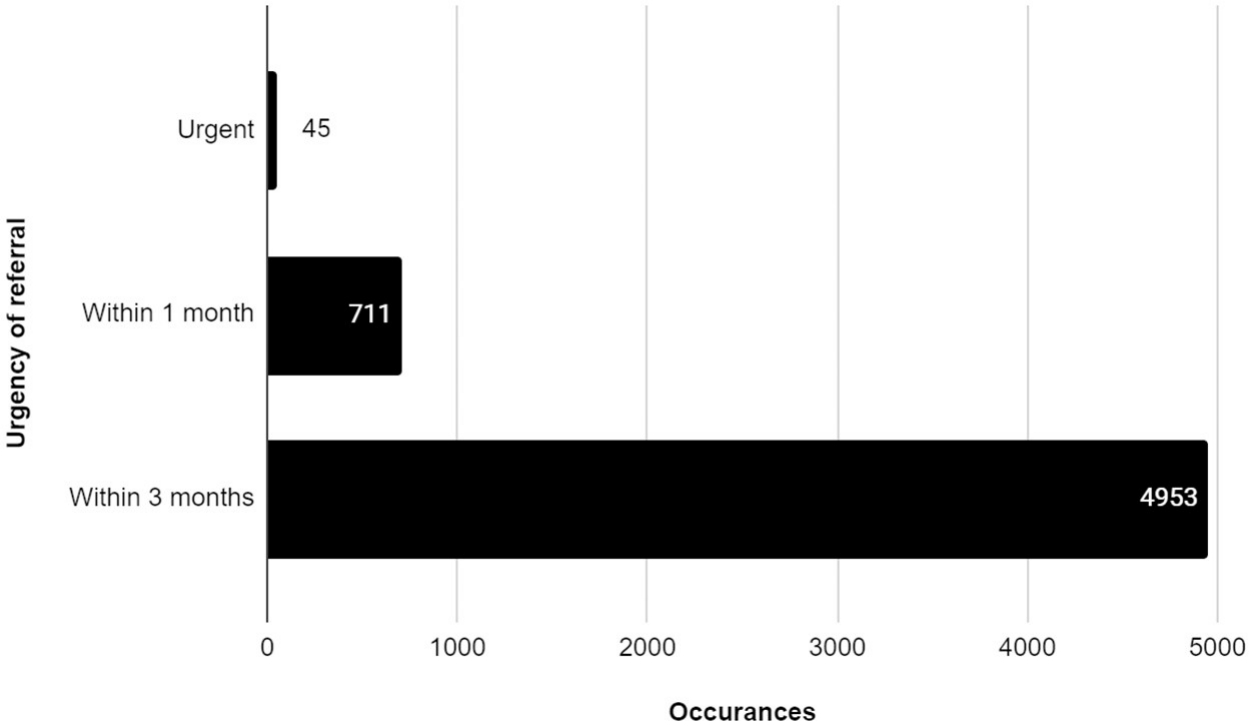
Urgency of referral to an eye care provider.

Regarding urgency of referral, less than 1% of referrals were considered urgent (0.8%; 45/5709), 12.5% of referred patients were to be seen within 1 month (711/5709), and 86.8% (4953/5709) were to be seen within 3 months of screening (Fig 4).

**Fig 5:**
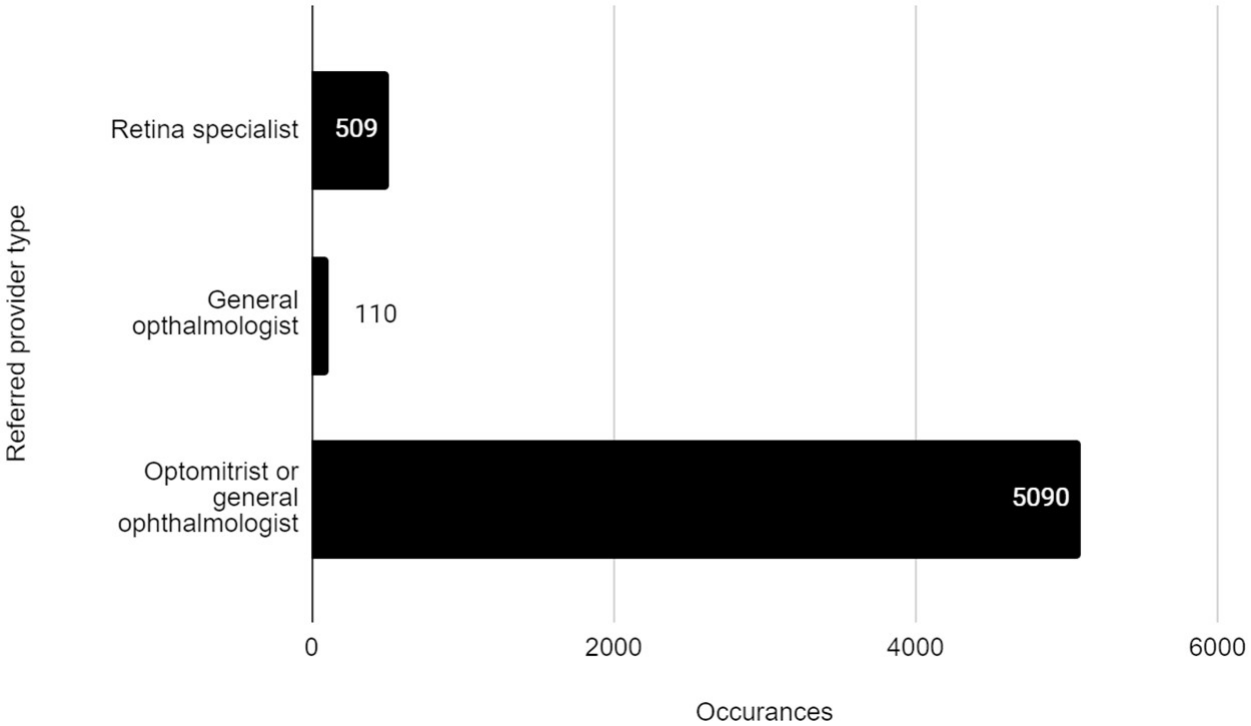
Referred provider types.

Of the 5709 referrals analyzed, 8.9% were to retina specialists (509/5709), and 1.9% (110/5709) designated general ophthalmologist as the appropriate provider type. The remaining were for any eye care provider (ophthalmologist or optometrist; 89.2%; 5090/5709) (Fig 5).

### Care continuity

During the 5-year timeframe analyzed, 2699 patients (26.8%) received two or more in-clinic screenings, accounting for 47.2% (6577/13,923) of all TDRS performed.

There was a significant difference in the proportion of referable DR pathology between single- and multiple-visit groups (12.9% and 24.3%, respectively; (*P* < .001)), suggesting that continuity of screening services doubled the odds of early detection of referable DR pathology (OR 2.2, 95% CI: 2.0–2.4; *P* < .001). Interestingly, the odds of future in-network screenings increased following the second TDRS encounter (OR 1.3, 95% CI: 1.2, 1.4; *P* < .001).

### Screening rates

**Fig 6:**
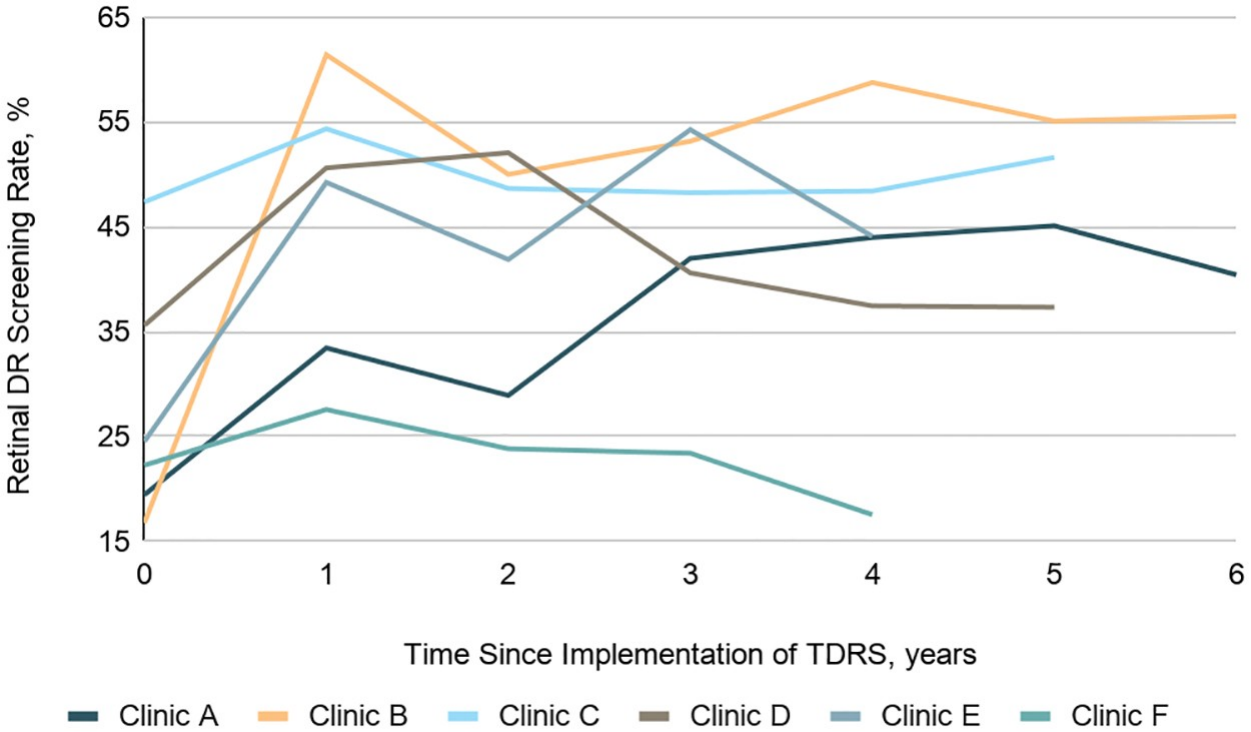
Comparison of diabetic retinopathy screening rates over time for adult diabetic patients among sampled clinics before and after implementation. Six audited ADEN clinics are shown, with time of TDRS program implementation represented by time 0 for all clinics. Actual implementation was on a rolling basis covering a 2 year period between February 2014 and February 2016. Abbreviations: DR, Diabetic retinopathy; TDRS, Telemedicine diabetic retinopathy screening.

DR screening rates increased in all audited clinics following TDRS implementation, though not all clinics sustained their initial gains (Fig 6). Screening rates in the year prior to implementation averaged 29.9% and ranged from 16.8% to 47.4%. The mean first-year screening rate increase was 18.5%, which, in a generalized estimating equation logistic regression model, doubled patients’ odds of being screened over the previous year (OR 2.2; 95% CI: 1.3–3.7; *P* = .003). Sustained screening rate gains averaged 14.0% (Table 5) and diabetic patients were 87% more likely to be screened for DR during the four years following implementation than they had been before TDRS implementation (OR 1.9; 95% CI: 1.1–3.1; *P* = .018).

**Table 5:** Annual rates of DR screening before and after TDRS program implementation.

	Diabetic patients, No./Total No., (%)		
Clinic	Pre-intervention^a^	Post-intervention^b^	Sustained increase in proportion screened, %
Clinic A	56/288 (19.4)	428/1155 (37.1)	17.6
Clinic B	65/388 (16.8)	861/1536 (55.1)	39.3
Clinic C	225/475 (47.4)	973/1954 (49.8)	2.4
Clinic D	183/514 (35.6)	888/1980 (44.9)	9.3
Clinic E	51/208 (24.5)	410/865 (47.4)	22.9
Clinic F	61/274 (22.3)	304/1329 (22.9)	0.6
**TOTAL**	**641/2147** (29.9)	**3864/8819** (43.8)	**14.0**

^a^Rate reported during the year prior to TDRS implementation.

^b^Average rate over first four years following TDRS implementation.

Abbreviations: DR, Diabetic retinopathy; TDRS, Telemedicine diabetic retinopathy screening.

### Patient satisfaction

Nine hundred fifty-two patients completed a TDRS satisfaction survey post-screening. Three quarters of those responding (73.2%; 522/713) were in their first decade since diagnosis, and approximately half (48.3%; 263/545) self-reported that their diabetes was well-controlled. Regarding TDRS, 92% (869/941) were satisfied with the screening experience; 82.5% (776/941) preferred the primary-care based TDRS service over visiting an eye care specialist; and 92.7% (868/936) would recommend TDRS to friends with diabetes. Similar large majorities rated the service convenient (94.5%; 885/937), comfortable (90.2%; 847/939), quick (94.0%; 877/933), and easy (93.8%; 879/937; Table 6). We observed a significant inverse correlation between duration of diabetes and HbA_1c_ control (*P* = .002), and a positive correlation between HbA_1c_ control and overall satisfaction with the TDRS experience (*P* = .02).

**Table 6:** Self-reported patient satisfaction with in-clinic TDRS experience.

Prompt	Responses	Agree or strongly agree	Proportion of responses favorable of TDRS, %
*I was satisfied with my eye exam experience*.	941	868	92.2
*I prefer this screening over visiting an eye doctor*.	941	776	82.5
*I would recommend this screening to friends with diabetes*.	936	868	92.7
*This exam was convenient*.	937	885	94.5
*This exam was comfortable*.	939	847	90.2
*This exam was quick*.	933	877	94.0
*This exam was easy*.	937	879	93.8

Abbreviations: TDRS, Telemedicine diabetic retinopathy screening.

## Discussion

DR screening rates in the US remain low due to patient-, provider-, clinic-, and system-level barriers. Many such barriers may be mitigated through convenient primary care-based telescreening [29,37], which has been shown to increase DR screening rates [21].

In this analysis of nearly fourteen thousand TDRS encounters during the first 5 years of an academic-community partnership of 22 FDSC sites serving rural and urban poor communities in Kentucky, the need for over 8000 specialist exams was eliminated; more than 2000 referable pathologies were detected; odds of patients’ being screened doubled, and gains were sustained over years; care continuity doubled the odds of early detection of referable pathology and increased odds of future screening compliance; and most patients preferred and endorsed the intervention over traditional specialist exams.

Regarding TDRS image gradeability, over ten thousand screenings adequate for detection of DR and other pathologies were performed, for a gradeability rate above 75%. This is just below the range observed in some similar studies of nFP screenings. For example, Gao et al., in a study of an urban primary care practice network with academic affiliations, observed a practical gradeability rate of 76.7% after accounting for both missing and inadequate images [38]. Chin et al., in comparing TDRS outcomes between a rural Native American Indian population and patients of an urban family practice identified 82.4 and 85.7% of images adequate for evaluation, respectively [39]. The inverse correlation between age and gradeability (observed here and corroborated elsewhere) [40] may explain some proportion of the ungradable images in this study, but given the wide range of rates over time and among clinics, we hypothesize that inconsistent image quality is at least partly due to multilevel barriers and inadequate implementation strategies that warrant further elucidation.

Diabetic pathology was found in 22% of adequate screenings. Other studies of TDRS programs in the US have reported similar DR rates [27,38,39,41–45], with rate variations likely tracking with variations in socioecological determinants [46].

More than 95% of screenings positive for DR were for non-proliferative DR (NPDR), suggesting an exceptional opportunity for providers to forestall disease progression before irreversible vision loss occurs. The proportion of adequately screened patients with proliferative DR (PDR; 1.0%, 95% CI: 0.8–1.2) was higher than that observed by Gao et al. (0.2%, 95% CI: 0.2–0.4) [38]. Evidence of diabetic macular edema (DME) was more common still (5.1%, 95% CI: 4.7–5.5), present in 23.0% of pathology-positive referrals. This DME rate is higher than that reported by Gao et al. (0.4%, 95% CI: 0.3–0.5) [38], but similar to rates observed by Gu et al. (4.4%, 95% CI: 2.4–7.4) and Varma et al. (3.8%, 95% CI: 2.7–4.9) [27,47]. These disparities may be the result of study population differences in diabetes duration, glycemic control, and screening compliance.

Almost 12% of adequate screenings revealed non-diabetic pathology, including intermediate stage or higher AMD and glaucoma. Such incidental findings of potentially disabling diseases increase the value of DR screening generally, and TDRS specifically, by reaching patients not otherwise receiving appropriate eye care.

Our observation that the odds for early detection of referable DR pathology were doubled by screening service continuity (OR 2.2, *P* < .001) reinforces both the value of the annual screening schedule and the importance of TDRS program sustainment. In concert, our observation that the odds of future in-network screenings increased following the second annual visit (OR 1.3, *P* < .001) suggests a small but significant cumulative effect of patient education and expectation on service utilization. Nevertheless, aggregate rates never approached the National Committee for Quality Assurance’s Diabetes Recognition threshold of 60%, pointing to higher level and upstream factors barring greater implementation effectiveness. Unfortunately, few studies of TDRS report follow-up screening rates over the large timeframe herein described [48], limiting opportunities for relevant comparison.

In contrast, a wide range of results have been published on the impact of TDRS for overall screening rates [42,49–53]. Of note, very few of these studies report on as large a network of clinics, or span both rural and urban poor populations, or surveille an equivalent timeframe. Taking our network in aggregate, the 1-year doubling of odds for being screened (OR 2.2; 95% CI: 1.3–3.7; *P* = .003) was similar to the increase described by Daskivich et al. for a safety-net system (OR 1.9; 95% CI: 1.3–2.9; *P* = .002) [54]. Our study also found a sustained pooled screening rate gain of 14% (95% CI: 11.7–16.1; *P* < 0.001) over four years, similar to the improvement observed by Jani et al. (15.8%; 95% CI: 13.0–16.6) [41], though that increase was reported only for the first year after implementation and regarded a smaller cohort of five clinics.

Despite large increases in screening rates for some of our clinics, others improved little. Further, gains were sustained in some clinics, whereas others’ performance regressed over time. These differences in TDRS adoption and sustainment reflect the challenges to implementation across disparate contexts and practices and point to unidentified upstream, multi-level variables affecting program success. Prior research on TDRS has mainly focused on patient-level factors influencing screening compliance, with scarce data on higher-level barriers.[55] Addressing this paucity, Liu et al. recently used content analysis methods with open-ended interviews of rural PCPs involved in TDRS delivery to identify several perceived provider-level barriers, including difficulty identifying when patients are due for screening, time constraints, and difficulty of referrals.[56] Barriers anecdotally observed during implementation of TDRS in a large urban safety-net system were reported by Ogunyemi et al. to include poor workflow integration, inadequate patient education, inconsistent image quality, and staff diversion.[57] Any, all, or none of these may explain the range of program outcomes we observed, and the identification of factors determining TDRS program success is a priority of our ongoing work.

One potential barrier that our patient survey data refutes is patient objection. Surveyed patients preferred TDRS to specialist exam and endorsed TDRS to friends, indicating a high patient valuation of the TDRS model [22].

Our study provides novel insights for state-scale TDRS implementation targeting underserved populations. To our knowledge, no other longitudinal studies in the US have described a TDRS network as geographically and administratively diverse, nor demonstrated sustained multi-year impact on screening rates, nor quantified patient-level effects of care continuity for odds of early detection and odds of future screening compliance. Combined, our findings provide a useful framework for other stakeholders to implement sustainable large-scale TDRS programs compatible with social distancing, appropriate for vulnerable populations, and synergistic with care continuity.

### Limitations

Though very few patients refused the TDRS service when offered, the studied pool was necessarily limited to those attending a network clinic for care, creating a potential selection bias by exclusion. Image quality, media opacities, and peripheral exclusion due to limited field of view each contributed to potential underestimates of pathologies.

The structure of our academic-community network prevented the efficient sharing of medical record data. The resulting paucity in demographic and clinical data is a limitation that precluded regression analysis to identify patient-level predictors of screening outcomes. Further, because most clinics served communities far from our academic system and referrals were therefore directed to outside specialists, we were unable to assess whether patients sought or received referred eye care—a target of our ongoing studies.

These limitations reflect the intrinsic nature of studies involving low-resource community partnerships. While they may limit generalizability, our study describes a strategy for large-scale TDRS implementation in FDSCs; advances understanding of outcomes of TDRS services for underserved populations; and lays the foundation for future research on barriers and facilitators of optimal TDRS implementation in the safety-net primary care setting.

## Conclusions

This report demonstrates that TDRS embedded in FDSCs can reduce the need for specialist appointments, increase patients’ odds of screening and early detection of vision-threatening pathology, and achieve sustained increases in screening rates for underserved populations. Despite high patient acceptability, screening rates across our clinics were variable and aggregate performance lagged the national DR screening rate, suggesting that to achieve full intervention potential, optimal implementation is essential.

Future studies should address poorly understood barriers to TDRS implementation at the level of professionals, healthcare institutions, and payer systems.

## Supporting information

S1 Dataset (CSV)
